# Deciphering the chronology of copy number alterations in Multiple Myeloma

**DOI:** 10.1038/s41408-019-0199-3

**Published:** 2019-03-26

**Authors:** Anil Aktas Samur, Stephane Minvielle, Masood Shammas, Mariateresa Fulciniti, Florence Magrangeas, Paul G. Richardson, Philippe Moreau, Michel Attal, Kenneth C. Anderson, Giovanni Parmigiani, Hervé Avet-Loiseau, Nikhil C. Munshi, Mehmet Kemal Samur

**Affiliations:** 1 Department of Data Sciences, Dana Farber Cancer Institute, Boston, MA 02215 USA; 2 Department of Biostatistics, Harvard T.H. Chan School of Public Health Boston, Boston, MA 02115 USA; 3 Inserm UMR892, CNRS 6299, Université de Nantes; Centre Hospitalier Universitaire de Nantes, Unité Mixte de Genomique du Cancer, Nantes, France; 4 Department of Medical Oncology, Dana Farber Cancer Institute, Harvard Medical School, Boston, MA 02115 USA; 5 VA Boston Healthcare System, Boston, MA 02115 USA; 6 University Cancer Center of Toulouse Institut National de la Santé, Toulouse, France; 7 Unité de Génomique du Myélome, IUC-Oncopole 2 Avenue Hubert Curien Cedex 1, Toulouse, 31037 France

## Abstract

Multiple myeloma (MM) and its precursor condition MGUS are characterized by chromosomal aberrations. Here, we comprehensively characterize the order of occurrence of these complex genomic events underlying MM development using 500 MGUS, and MM samples. We identify hyperdiploid MM (HMM) and non-HMM as genomically distinct entities with different evolution of the copy number alterations. In HMM, gains of 9,15 or 19 are the first and clonal events observed as clonal even at MGUS stage. These events are thus early and may underlie initial transformation of normal plasma cells to MGUS cells. However, CNAs may not be adequate for progression to MM except in 15% of the patients in whom the complex subclonal deletion events are observed in MM but not MGUS. In NHMM, besides the driver translocations, clonal deletion of 13 and 1q gain are early events also observed in MGUS. We combined this information to propose a timeline for copy number alteration.

## Introduction

Multiple myeloma (MM) is a genetically complex disease characterized by an abnormal proliferation of clonal plasma cells (PCs)^1–3^. MM is characterized by both clinical and genomic heterogeneity^4^. The copy number alterations (CNA) are one of the most prominent genomic perturbations in MM reported using cytogenetics and fluorescent in situ hybridization (FISH)^5–7^. CNAs are characterized by the trisomy of certain odd number chromosomes 3, 5, 7, 9, 11, 15, 19, 21 in a proportion of patients (hyperdiploid MM, or HMM); while the other nonhyperdiploid MM (NHMM) patients typically display chromosomal rearrangements especially involving the IgH locus^8,9^ in some cases. Also associated with hemizygous deletion of chromosome 13^5,10–16^.

Malignant transformation of normal plasma cell into MM cell occurs at sequential clinical phases starting with monoclonal gammopathy of undetermined significance (MGUS), which is the first and essential stage of the disease and critical for the full understanding of clonal evolution. MGUS is followed by smoldering multiple myeloma (SMM), an asymptomatic clonal plasma cell disorder^17,18^, and eventually by MM. MGUS is found in about 3% of individuals over 50 years of age and has a very low progression rate^19,20^. These stages are creating an opportunity to study the evolution process. Although aneuploidy is a hallmark of MM, no previous study has considered the sequence of occurrence of these complex genomic events during MM development. In view of the complex MM genome heterogeneity, several clonal evolution paths for MM have been proposed^21,22^ including linear and branching evolution. Spatial sequencing and single-cell sequencing studies also showed that early stages could be driven by unique events followed by regional evolution in advanced disease^23,24^. These proposed models were either focused on single nucleotide variants or used paired samples from diagnosis and relapse.

Here, we use the largest available genomic dataset that covers the progression stages in MM, to characterize when and in what sequence each CNAs occur in MM. We infer the order of CNA events at diagnosis from cross-sectional data and validate our results using early-stage samples from MGUS patients to identify how CNA clonal patterns change between stages in hyperdiploid and non-hyperdiploid MM to propose a model that explains the order of copy number changes in MM and identify common mechanisms between the two subgroups.

## Methods

### Study samples

The study involved the use of human samples, which were collected after written informed consent in accordance with the Declaration of Helsinki. The patients were collected from different centers from the Intergroup Francophone du Myélome, but all the samples were sent to one central laboratory for preprocessing and DNA profiling with Affymetrix Cytoscan HD SNP array. We collected 336 newly diagnosed MM samples from the IFM/DFCI 2009 clinical trial (Clinical Trial Number: NCT01191060), and 164 MGUS from IFM2008/02 study. IFM/DFCI was a Phase III, multicenter, randomized, open-label study designed to evaluate the clinical benefit from the drug combination RVD without immediate high-dose therapy (HDT) followed by lenalidomide maintenance versus RVD plus HDT and ASCT followed by lenalidomide maintenance. After bone marrow collection from all patients at all stages, CD138+ selection was performed on purified myeloma cells from bone marrow. All patient samples with symptomatic and progressive multiple myeloma based on International Multiple Myeloma Working Group criteria were collected at diagnosis. MGUS samples were diagnosed based on clinical diagnostic criteria set forth by the International Myeloma Working Group (IMWG). The diagnoses were confirmed by either serum/urine protein electrophoresis, immunofixation and light-chain assays; or immunohistochemistry analyses of the bone marrow biopsy, or a combination of these tests.

### FISH analysis

For all other samples, 100,000 plasma cells were stored for FISH analyses, and remaining plasma cells were stored in RLT Plus buffer (Qiagen, Paris, France) for subsequent DNA and RNA extraction. Sorted plasma cells were fixed in Carnoy’s fixative and stored at −20 °C until hybridization. After slide preparation, they were denatured in 70% formamide for 5 min, dehydrated in 70%, 85%, and 100% ethanol series. The probes specific for the t(4;14), t(11;14) and t(14;16) were purchased from Abbott Molecular and denatured separately for 5 min at 75 °C. After denaturation, the probes were dropped on the plasma cells and hybridized overnight at 37 °C. Then, coverslips were removed, and the slides were washed for 2 min in 2xSSC-0.1% Triton at 75 °C. For patients who did not have t(11;14) FISH results, we used RNAseq data to impute the t(11;14) status.

### SNP array analysis

Genomic DNA purified from primary MM and MGUS samples was interrogated with Affymetrix CytoScan HD Arrays (Affymetrix, Santa Clara, CA) according to the manufacturer’s instructions. CEL files were generated from scanned array image files by Affymetrix GeneChip Command Console software were imported into Chromosomal Analysis Suite software. Copy number data files (CYCHP files) were generated using ChAS Analysis Files for CytoScan HD Array hg19 as a reference.

### Clonality analysis

We have investigated divergence in clonal lineages forming distinct subpopulations, resulting in intratumor heterogeneity (ITH)^25^ both at precursor state and at diagnosis of MM^26^ by evaluating clones (early events) and sub-clones (sub sequent events) using Cytoscan HD array data. To assess copy number abnormalities for each cytoband and sample we first calculated a smooth signal using Affymetrix ChAS with default settings. Smoothed CN estimates for each probe set were exported from CYCHP files using Affymetrix ChAS. Smoothed copy number signals are reducing the noise by applying certain filters and are used to detect edges for gains and deletions^27^. Next, we downloaded cytoband locations for the hg19 reference genome from the UCSC Genome Browser website (http://hgdownload.cse.ucsc.edu/goldenPath/hg19/database/). Sex chromosomes were excluded from our analysis. Probe sets were assigned to cytobands by coordinates. CN estimates were calculated as the average CN of all the probe sets within each cytoband region. A “clonality scale” for each gain or deletion within each cytoband was then calculated as the normalized absolute deviation (range: 0 to 1) of cytoband CN from the diploid state, to analyze gains and deletions jointly. All cytobands in each chromosomal arm were then used to get clonality distributions. Chromosomal arms were ordered by mean clonality estimate starting from highest to the lowest. One-way ANOVA with groups defined by chromosomal arms, and Tukey HSD test was used to test for differences between the means of consecutive cytoband clonality levels, using the following stepwise method. If the null hypothesis, the mean is the same for all cytoband levels, was rejected, then Tukey HSD’s results were used to define discrete clonality levels. Five clonality levels were determined to start from the chromosomal arm with the highest mean clonality. All chromosome arms that are not significantly different (Tukey HSD *p*-value > 0.05) from the chromosome arm with the highest mean clonality were grouped. Whenever the step reaches a significant difference (Tukey HSD *p*-value ≤ 0.05) between chromosome arm with the highest mean in the group, the compared arm was assigned to next clonality level as the top node and the following cytobands were compared against it. This iteration continues until all chromosome arms are assigned to clonality levels. Because the samples were normalized separately, this allowed us to characterize clonality independent from purity status (Supplementary Figure 1).

Clonality levels for each CNA are shown in oncoplots created based on the R package *Maftools*. Only CNA’s with 10% or more frequency were kept, and samples were clustered using Hierarchical Agglomerative Clustering (Ward.D2) with Manhattan distance. CNA timing and confidence intervals in HMM and NHMM are calculated with bootstrap with 1000 iterations using R package *boot*.

### Statistical analysis

All statistical and downstream analysis were carried out using R (v3.3.2). The *pheatmap, FSA, plot3D, corrplot, proxy, maftools, ggplot2, ggscatter* and *boot* packages were used. To characterize the patients and events in the study, we used median (IQR) and frequency (%). The Fisher test was used to assess associations of event occurrence between older (age ≥ 60) and younger patients (age < 60).

To compare differences in the frequency of events between H-MM and NH-MM by stage, the Wilcoxon rank-sum test was used. The percentage of clonal events between two stages (MGUS and MM) were compared using Fisher test. FDR (Benjamini–Hochberg) method was calculated using R. Spearman correlation was used to measure correlation in clonality index is observed for each of the events between all HMM samples and those without tetraploidy patients.

## Results

### Copy number alterations in MM

We studied myeloma cells from 336 newly diagnosed patients and identified a significant number of CNAs affecting either whole chromosomes or an entire chromosome arm using smoothed averages of copy number data from Affymetrix Cytoscan HD arrays. As shown in Supplementary Figure 2a, 183 of 336 (54.5%) MM patients were classified as HMM with gains of three or more chromosomes. The remaining 153 (45.5%) were classified as NHMM. The proportion of HMM cases increased with age, 62% patients older than 60 years of age having HMM compared to 46% of patients younger than 60 years of age (*p* = 0.004).

**Fig. 1:**
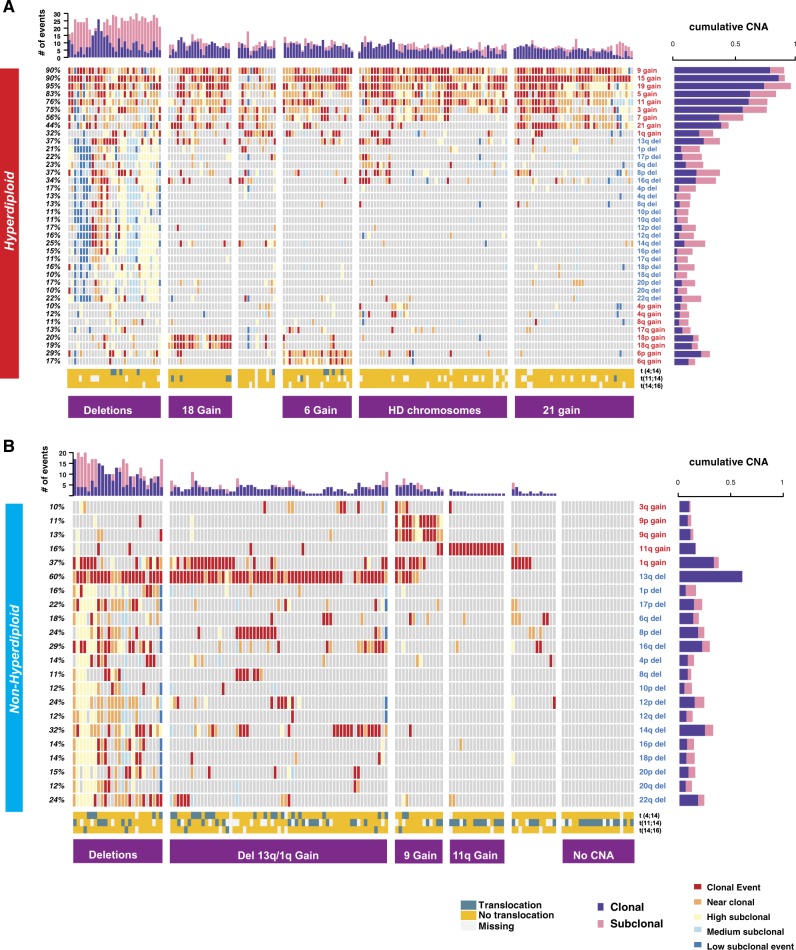
Clonality and co-occurrence of copy number alterations in MM. Oncoplots showing the clonality level for CNAs (rows) in each patient (columns) at diagnosis for two aneuploidy groups: **a** HMM and **b** NHMM. The clonality level of each event is categorized from clonal (dark red) to low subclonal (dark blue). Horizontal bars on the right indicate the proportion of clonal (purple) and all-subclonal-combined (pink) events for each alteration. Bar plots on top show the total number of genomic events for each patient; again, clonal events are purple, and subclonal events are pink. The bottom three rows annotate translocation detected by FISH (blue, yellow, and white colors show detected translocations, no translocation and missing data, respectively). Our classifications of HMM and NHMM patients into subgroups based on copy number patterns are indicated at the bottom in the purple boxes

As several CNAs in both HMM and NHMM are exclusive to each group, suggesting distinct driver influences, the two groups were further analyzed separately for the rest of the study. We investigated the frequency and the order of copy number alterations in each HMM sample, using smoothed signals from Cytoscan HD profiles, and merging p and q arms for eight odd number chromosomes 3, 5, 7, 9, 11, 15, 19, 21. In HMM, gains in chromosome 19 (95%), 15 (90%) and 9 (90%) are the most frequent events, followed by gains in other odd number chromosomes 5,11,3,7 and 21 (Fig. 1a). Del13 is the most common deletion event detected in 37% of the HMM patients. The majority of the remaining events in HMM, at a lesser frequency (10–21%), are deletions. More than 96% of HMM samples had concurrent gains in at least two of the three most frequent chromosomes (9, 15, and 19). The NHMM group showed del13 as the most frequent event (60%) followed by 1q gain (37%) and 14q deletion (32%) (Fig. 1b). 14% of NHMM patients had no identifiable recurrent (i.e. observed in more than 10% of patients) CNAs. In general, HMM patients tend to have more events [median = 10, IQR = [8–12]] compared to NHMM patients [median = 3, IQR = [1–5]] (*p* < 2.2e−16) (Fig. 1a, b upper bars).

### Clonality of the genomic events in HMM and NHMM

We next reconstructed the order of the copy number alterations in each sample by estimating the clonality of each CNA. Clonal events are considered an early event while subclonality defines later changes. To understand the order of occurrence of each chromosomal change, we resolved the clonality of each gain or loss events in five categories which we term clonal (including clonal and near-clonal), or subclonal (including high, medium, and low) (Fig. 1a, b). Clonality segments defined by the Tukey HSD were used to define discrete clonality levels. Five clonality levels were determined to start from the chromosomal arm with the highest mean clonality. The clonality level of each event is categorized from clonal (dark red) to low subclonal (dark blue). This is explained in detail in the Clonality Analysis section in Methods. In HMM, the gain of chromosome 15 was the most frequent clonal event, observed in 86% of patients (Supplementary Figure 3) followed by chromosome 9 in 78% of the patients. Surprisingly, although chromosome 19 gain is the most frequent event overall, its clonal occurrence was lower (73%) than clonal chromosome 15 gain. 86% of HMM patients had concurrent clonal gains of at least two out of three most frequent chromosomes (9, 15, and 19). Moreover, less frequent events such as chromosome 21 gain, 18p gain, and 1q gain showed higher frequency of clonal occurrence compared to other events indicating that when these events occur, they are early events. (Supplementary Figure 3). The majority of deletions occur as late subclonal events with few or none observed as clonal events (Supplementary Figure 3).

In the NHMM group, three events, del13, gain of 1q and gain of 11, had the highest frequency of clonal occurrence. Gain of 11, although detected only in 15% of NHMM patients (Fig. 1b), was clonal in 92% of cases (Supplementary Figure 3); del13, observed in 60% of NHMM patients, was clonal in 99% of them. Interestingly, 13% of NHMM patients also had chromosome 9 gain. Most were clonal events signifying their importance in the early stages of the disease.

We next investigated the co-occurrence of copy number events, to understand sequential genomic changes that may underlie myelomagenesis. We first applied a clustering approach with estimated event orders to help define subgroups in both HMM and NHMM. In HMM, co-occurrence of gains of chromosome 6 or 18 identified two distinct subgroups. A third group, comprising about 17% of HMM patients, showed multiple deletions with similar level of subclonality for each deletion suggesting a possible common temporal and etiological relationship between these copy number losses (Fig. 1a). The largest hyperdiploid group is characterized by gains of odd-numbered chromosomes but separated by presence or absence of gain of chromosome 21. Lastly, a small cluster between those characterized by gains of chromosomes 18 and 6, appears to be missing major hyperdiploid events but is enriched in gain of 1q.

In the NHMM group, clusters were driven by myeloma with 11 or 9 gain, with del13 and a group without any chromosome-level copy number changes. Gain of 11 was mutually exclusive from other events (Fig. 1b). The fourth group (del 13) showed enrichment in subclonal deletion in a series of chromosomes very similar to those observed in the deletion group in HMM. Importantly, the majority of NHMM patients had one of the IgH-related translocations which have been described to be clonal, but their occurrence in HMM is very infrequent (Fig. 1a). Associations between individual events were also confirmed with Jaccard Index (Supplementary Figure 4).

**Fig. 2:**
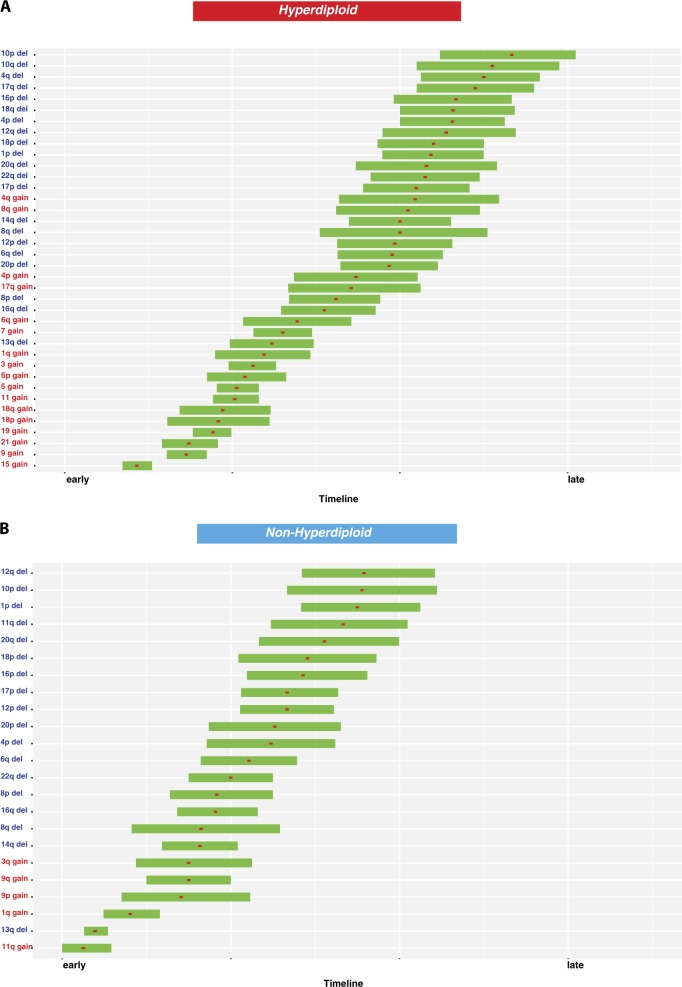
Timeline in MM. CNA timing interval in HMM (**a**) and NHMM (**b**) are calculated with bootstrap with 1000 iterations using the R v (3.3.2) package *boot*. Timing on the *x*-axis is from early to late, early being 1 and late being 4 on clonality score. Red squares show the mean timing for each event (*y*-axis). Horizontal bars indicate 90% bootstrap confidence interval of the mean

Synthesizing the data to evaluate the occurrence of the sequence of CNA events in HMM, the gains of chromosome 15, 9, 21, 19, and 18, when present, are the first events in MM (Fig. 2a). In fact, 92% of all HMM patients have two and 71% have three of these chromosomes involved in trisomies. These are followed by the occurrence of trisomies of 11, 5, and 6 followed by trisomies of 3 and 7. Not all these trisomies are required and as seen in Supplementary Figure 5, combinations of 2, 3, or more chromosomes may be adequate as early events in the myelomagenesis. For NHMM samples, deletion 13 and 11 gain, when present, are early events followed by 1q or Ch 9 or 3q gain. The majority of deletions, in both groups, are subclonal and hence late events (Fig. 2b).

### Clonality of genomic events in MGUS

As all myeloma originate from their precursor conditions, the alterations we identified as clonal and likely early in MM, must also exist in MGUS. To confirm these observations in MM, we analyzed CNAs in purified plasma cells from 164 MGUS patients.

**Fig. 3:**
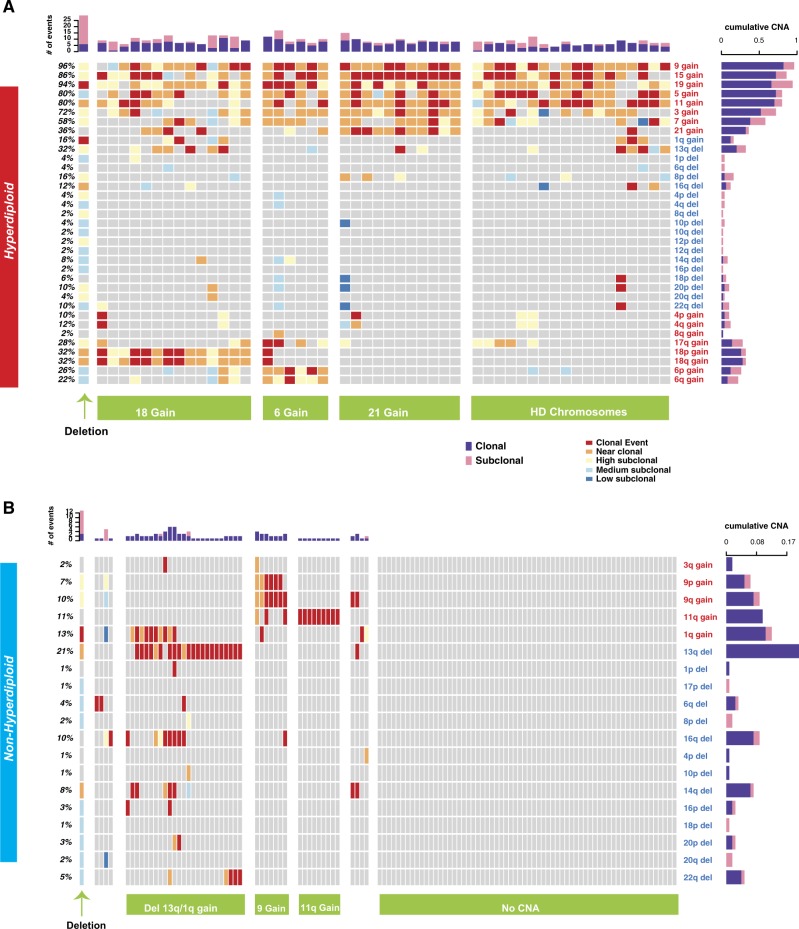
Clonality of copy number alterations in MGUS. **a**, **b** Oncoplots showing the clonality level for CNAs (rows) in each patient (columns) in MGUS for hyperdiploid (**a**) and nonhyperdiploid samples (**b**). Clonality level of each event is shown from clonal (dark red) to low subclonal (dark blue). Horizontal bars on the right indicate the proportion of clonal (purple) and all-subclonal-combined (pink) events for each alteration. Bar plots on top show the total number of genomic events for each patient; again, clonal events are purple, and subclonal events are pink. Our classifications of HMM and NHMM patients into subgroups based on copy number alterations are indicated at the bottom

Fifty of 164 (30.5%) MGUS patients were classified as hyperdiploid (HMGUS). As seen in Supplementary Figure 6A, as in MM, CNAs were also observed at the MGUS stage. Similar to symptomatic MM, gains in chromosomes 19 (95%, 94%), 15 (86%, 86%), and 9 (87%, 96%) are the most frequent events at MGUS stage (Fig. 3a, b, Supplementary Figure 6A). The nonhyperdiploid group showed del13 as the most frequent event (21%) followed by the gain of 1q (13%) in MGUS (Fig. 3a, b, Supplementary Figure 6B). To define the co-occurrence of copy number events in MGUS, we next clustered hyperdiploid MGUS samples using the frequently observed events in MM. The groups with gains of chromosomes 6, 18, or 21 were observed at MGUS (Fig. 3a, b). Nonhyperdiploid subgroups were also consistent between early and late stages (Figs. 1b and 3a, b) except for complex deletions.

**Fig. 4:**
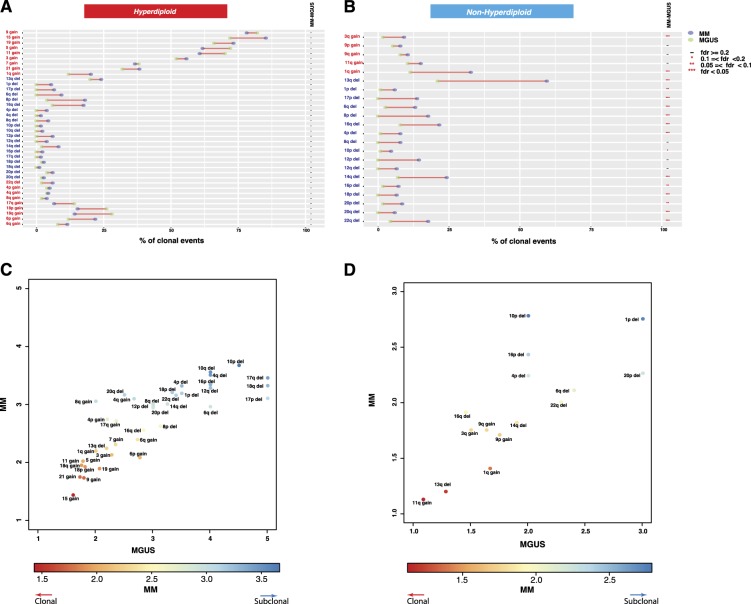
Clonality indices of copy number events in MGUS and MM. **a**, **b** Comparison of clonal CNA frequencies between MGUS and MM for HMM (**a**) and NHMM (**b**). Rows represent chromosome arms and are in the same order in Fig. 2. The frequencies of events in MGUS and MM are represented by green and purple circles, respectively. Adjusted significance levels for each comparison (FDR) are given in the rightmost columns. **c**, **d** 2D scatterplots of the clonality index, computed as the mean clonality score when the alteration is detected. The score is between one and five, with one being clonal and five being low subclonal. Each point represents a chromosome arm event. Axes represent stages: MGUS (*x*-axis) and MM (*y*-axis). We present separate scatterplots for hyperdiploid (**c**) and nonhyperdiploid (**d**) samples

The clonal CNAs observed in HMM are also observed at a similar frequency in HMGUS (Fig. 4a) confirming the acquisition of these genomic changes early in the disease process. Interestingly, the majority of subclonal deletions (deletions targeting 1q, 6q, 8p, 12p, 12q, 14q, 16p, 16q, and 17p) observed in both HMM and NHMM patients (Fig. 4b) were not observed in MGUS, suggesting late occurrence of these events.

### Acquisition of copy number events from MGUS to MM

To further investigate the sequence of copy number alterations, we next calculated in HMM and NHMM an average clonality score for each chromosomal alteration using a 1 to 5 clonality index (1 being clonal 5 being low subclonal). In the HMM, 38 events were plotted (Fig. 4c) in a two-dimensional space. Early and clonal events such as the gain of chromosomes 15, 21, 9, and 19 were clustered together with lowest (closest to clonal) average scores. These events are followed by gain of 18, 11, and 5. Del13 and gain of 1q are in the next segment with the gain of chromosomes 3, 7, and 6. The majority of deletion events including high-risk markers 1p, 17p, were clustered in the outer part indicating their late occurrence. In NHMM, del13 and 11 gains were the initial events at all stages followed by the gain of 1q, trisomy 9 and deletion 14 (Fig. 4d). As in HMM, the NHMM group also showed the majority of deletions including deletion 1q and 17p as late subclonal events.

## Discussion

We have studied CNAs in myeloma and validated the observed evolution of CNAs utilizing MGUS samples. Our analysis clearly identifies HMM and NHMM as genomically distinct entities with significant differences in the acquired chromosomal alterations. We do identify overlapping changes between the two groups, such as del13, 1q gain and complex deletions observed in a proportion of patients in both subtypes. Although CNAs have been described in the literature using FISH or genomic profiling^28,29^, this study for the first time describes clonality estimates rather than absolute copy number changes and relative timing of CNAs. Moreover, currently there is no study systematically and carefully evaluating the chronology of all copy number alterations from precursor stages to MM which would provide insight into the MM development and progression.

A major finding from our study is that the observed clonal CNAs in HMM are also clonal at the MGUS stage, suggesting that these events are early and may underlie initial transformation of normal plasma cells to MGUS cells. These changes may not be sufficient to provide the required proliferative capacity for MM; however, they do lead to clonal expansion of the plasma cells. In 15% of the patients in whom the complex deletion events observed in MM but not at MGUS stage may account for the changes responsible for progression to MM, but in the majority of the patients, CNAs may not be adequate for progression to MM. From this analysis, we can postulate that further mutational and/or epigenomic changes may drive progression in these patients.

**Fig. 5:**
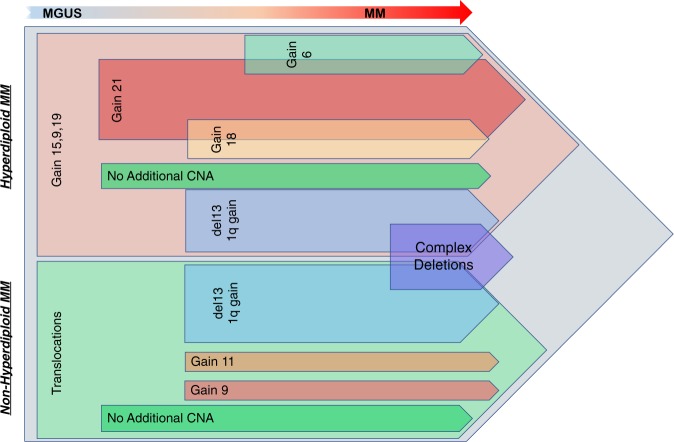
Evolution of copy number alterations during MM development. Proposed timeline of acquisition of CNVs during evolution from MGUS to MM

We have reconstructed a model representing the timeline of CNAs in MM development using combined clonality estimates with co-occurrence analysis from all stages of plasma cell disorders (Fig. 5). The reconstruction suggests five major copy number paths for HMM (each path is given with different colors in Fig. 5 for HMM and NHMM). Initial gains of 9, 15 or 19; with the clonal occurrence of at least 2 of these observed in 86% of HMM patients. In fact, chromosomes 9 and 15 are together in 69% of MM patients and may play the greatest role in the initiation of MGUS transformation where they are observed together in 64% of the patients. These changes are followed by gains in one of the chromosomes 6, 18, or 21; gain of 21, when present, is a very early event and occurrence of gain of 6 and 18 is mainly mutually exclusive. A group of HMM patients alternatively acquire deletion 13 and/or 1q gain and a subset of these samples will eventually get complex deletions. Surprisingly 20% of HMM samples do not acquire any new CNAs after the initial events. Our results clearly demonstrate that not all trisomies are required or occur at the same time. Similar to trisomies, tetraploidy is also observed in MM in around 10% of cases at diagnosis^30^. We have corrected our clonality estimates incorporating the tetrasomy data to overcome any bias introduced by tetrasomy cases (~15% of HMM group patients at diagnosis in our cohort). As seen in Supplementary Figure 7, a high correlation (*r* = 0.96) in clonality index is observed for each of the events between all HMM samples and those without tetraploidy patients, suggesting that our estimates are accurate and are not biased by the presence of tetraploidy. NHMM, on the other hand, is well known to have clonal IgH-associated translocation events as an initiating feature which is also observed in MGUS. These translocations are well described to be clonal at each on these stages. However, unlike HMM, the NHMM group shows only a few CNAs at an early stage and does not accumulate frequent additional alterations. These results suggest the possibility that the trisomies provide a similar molecular change as translocation event. The only exception to this observation is the deletion group that involves copy number loss for 15 or more chromosome arms. Interestingly, for each patient with these deletions, the level of subclonality was very similar for each of the deletions suggesting the occurrence of larger catastrophic genomic events affecting all the deletion chromosomes at the same time. This suggests that copy number events in MM would follow a punctured evolutionary process. This would also suggest a greater degree of genomic instability in the complex deletions group.

We have carefully considered the possibility that in MGUS samples the clonal population may be partly contaminated by CD138+ normal polyclonal plasma cells affecting the proportion of the true clonal population. This may possibly affect the nonhyperdiploid arm of MGUS, with a significant fraction of samples with no CNA being assigned to this group. However, our method to estimate clonality partly overcomes this problem. Additionally, for those changes which are clonal, we should at least observe subclonal level of CNAs in MGUS despite the presence of a significant proportion of non-clonal plasma cells. So, the total absences of CNAs in a subgroup would at least rule out the presence of clonal or near clonal plasma cell population. Observing the same complex deletions in both HMM and NHMM arm also suggest that a similar mutational process may be operative to induce such deletions irrespective of initial events.

Our results also highlight that for both the HMM and NHMM groups the major copy number events are not sufficient for eventual malignant transformation since only a small fraction of MGUS patients progress to MM. However, complex deletions not observed in MGUS may help us with early diagnosis of MM in the subset of patients. During MM development many somatic alterations other than CNA also occurs. Integrating information from the acquisition of other somatic alterations can identify additional driver events and improve our ability to predict MM progression.

In conclusion, here we have described the timeline of initial copy number alterations observed in MM and confirmed their early occurrence using data from a unique early stage plasma cell disorder case. Similarities between stages show that large scale DNA alterations happen early, however, some copy number hotspots are enriched over the time which could be important for disease progression. Studying these regions with larger cohorts may provide additional information on biomarker discovery and new therapeutic target discovery.

## Supplementary information

Supplementary Figure Legends

Supplementary Figure 1

Supplementary Figure 2

Supplementary Figure 3

Supplementary Figure 4

Supplementary Figure 5

Supplementary Figure 6

Supplementary Figure 7

Supplementary Table 1

Supplementary Table 2

## References

[1] Greipp PR (2005). International staging system for multiple myeloma. J. Clin. Oncol..

[2] Kyle RA (2003). Review of 1027 patients with newly diagnosed multiple myeloma. Mayo Clin. Proc..

[3] Morgan GJ, Walker BA, Davies FE (2012). The genetic architecture of multiple myeloma. Nat. Rev. Cancer.

[4] Munshi NC, Avet-Loiseau H (2011). Genomics in multiple myeloma. Clin. Cancer Res..

[5] Avet-Loiseau H (2009). Prognostic significance of copy-number alterations in multiple myeloma. J. Clin. Oncol..

[6] Samur MK (2013). The shaping and functional consequences of the dosage effect landscape in multiple myeloma. BMC Genom..

[7] Bolli, N., et al. Analysis of the genomic landscape of multiple myeloma highlights novel prognostic markers and disease subgroups. *Leukemia.* (2017). 10.1038/leu.2017.344. 10.1038/s41375-018-0037-9 PMC6092251 29789651

[8] Robiou du Pont S (2017). Genomics of multiple myeloma. J. Clin. Oncol..

[9] Corre J, Munshi N, Avet-Loiseau H (2015). Genetics of multiple myeloma: another heterogeneity level?. Blood.

[10] Bochtler T (2011). Hyperdiploidy is less frequent in AL amyloidosis compared with monoclonal gammopathy of undetermined significance and inversely associated with translocation t(11;14). Blood.

[11] Chng WJ (2007). Molecular dissection of hyperdiploid multiple myeloma by gene expression profiling. Cancer Res..

[12] Chng WJ (2005). A validated FISH trisomy index demonstrates the hyperdiploid and nonhyperdiploid dichotomy in MGUS. Blood.

[13] Chretien ML (2015). Understanding the role of hyperdiploidy in myeloma prognosis: which trisomies really matter?. Blood.

[14] Li Y (2013). Classify hyperdiploidy status of multiple myeloma patients using gene expression profiles. PLoS One.

[15] Prideaux SM, Conway O’Brien E, Chevassut TJ (2014). The genetic architecture of multiple myeloma. Adv. Hematol..

[16] Chng WJ (2006). Prognostic factors for hyperdiploid-myeloma: effects of chromosome 13 deletions and IgH translocations. Leukemia.

[17] Rajkumar SV, Kyle RA, Buadi FK (2010). Advances in the diagnosis, classification, risk stratification, and management of monoclonal gammopathy of undetermined significance: implications for recategorizing disease entities in the presence of evolving scientific evidence. Mayo Clin. Proc..

[18] Rajkumar SV, Landgren O, Mateos MV (2015). Smoldering multiple myeloma. Blood.

[19] Kyle RA (2006). Prevalence of monoclonal gammopathy of undetermined significance. N. Engl. J. Med..

[20] Kyle RA (2007). Clinical course and prognosis of smoldering (asymptomatic) multiple myeloma. N. Engl. J. Med..

[21] Manier S (2017). Genomic complexity of multiple myeloma and its clinical implications. Nat. Rev. Clin. Oncol..

[22] Bolli N (2014). Heterogeneity of genomic evolution and mutational profiles in multiple myeloma. Nat. Commun..

[23] Rasche L (2017). Spatial genomic heterogeneity in multiple myeloma revealed by multi-region sequencing. Nat. Commun..

[24] Lohr JG (2016). Genetic interrogation of circulating multiple myeloma cells at single-cell resolution. Sci. Transl. Med..

[25] Davis A, Gao R, Navin N (2017). Tumor evolution: linear, branching, neutral or punctuated?. Biochim. Biophys. Acta Rev. Cancer.

[26] Kominami R (1990). A sensitive assay for detecting mutations resulting from unequal homologous recombination without phenotypic selection. Mutat. Res..

[27] Yavas G, Koyuturk M, Ozsoyoglu M, Gould MP, LaFramboise T (2009). An optimization framework for unsupervised identification of rare copy number variation from SNP array data. Genome Biol..

[28] Lopez-Corral L (2011). The progression from MGUS to smoldering myeloma and eventually to multiple myeloma involves a clonal expansion of genetically abnormal plasma cells. Clin. Cancer Res..

[29] Mikulasova A (2016). Genomewide profiling of copy-number alteration in monoclonal gammopathy of undetermined significance. Eur. J. Haematol..

[30] Sidana S (2019). Rapid assessment of hyperdiploidy in plasma cell disorders using a novel multi-parametric flow cytometry method. Am. J. Hematol.

