# Supplementary material for: Deciphering the chronology of copy number alterations in Multiple Myeloma
Source: Blood Cancer J. 2019 Mar 26;9(4):39. doi: 10.1038/s41408-019-0199-3 (PMC6435669; doi:10.1038/s41408-019-0199-3)
Supplement: Supplementary file 1 — Supplementary Figure Legends [file 41408_2019_199_MOESM1_ESM.docx]

**Supplementary Figure Legends**

**Supplementary Figure 1. Determination of clonality levels for CNAs**. Box plot shows clonality distribution of cytobands within chromosome arms (p and q separately) for newly diagnosed MM sample. The y-axis shows clonality levels (range 0 – 1[clonal]) and chromosome arms are ordered from 1p to 22q on the x-axis. Horizontal lines show clonality segments defined by the Tukey HSD test. Events between the red and orange lines are classified as clonal, values between the orange and yellow lines are classified as near clonal and so on**.**

**Supplementary Figure 2. Summary of copy number alterations (CNAs) in MM**. **A)** Heat map of smoothed copy number estimates for 336 newly diagnosed MM patients. Columns represent patients and are clustered by Hierarchical Agglomerative Clustering (Ward.D2) with Euclidean distance using the R (v3.3.2) package *pheatmap*. Rows represent chromosome arms and are arranged from 1p to 22q from top to bottom. Copy number gains are indicated with red colors; deletions with blue colors, with intensity depicting copy number as indicated in the legend. The top three rows annotate translocations detected by FISH: blue indicates translocation, yellow no-translocation, and white missing data. **B)** Frequencies of copy number alterations in HMM and NHMM groups. The positive portion of the vertical scale is frequency of gains, while the negative portion is minus the frequency of deletions. The positive and negative portions are also colored red and blue respectively.

**Supplementary Figure 3. Relative frequency of CNAs in MM, and MGUS for hyperdiploid and non-hyperdiploid samples**. For each CNA, the relative clonality frequency is calculated as the number of samples at a particular clonality level, divided by the total number of samples that carry the CNA. Clonality levels are shown with red (clonal), orange (near clonal), yellow (high subclonal), light blue (medium subclonal) and dark blue (low subclonal). CNAs in each plot ordered by clonal event frequency (red bars).

**Supplementary Figure 4. Jaccard similarity coefficients between genomic events for MM samples in HMM and NHMM groups.** Size and color of circles represent the magnitude of the Jaccard index. Bigger circles indicate a higher index. Colors are depicted in the legend on the right.

**Supplementary Figure 5. Heat map of clonal copy number gains for early events in HMM.** Clonal copy number gains are indicated with red colors as indicated in the legend. Samples are clustered by Hierarchical Agglomerative Clustering (Ward.D2) with Manhattan distance using the R (v3.3.2) package *pheatmap*. The top three rows annotate translocations detected by FISH: blue indicates translocation, yellow no-translocation, and white missing data.

**Supplementary Figure 6. Copy number alterations for MGUS samples. A)** The heat map shows smoothed copy numbers for MGUS samples. Columns represent patients and are clustered by Hierarchical Agglomerative Clustering (Ward.D2) with Euclidean distance using the R (v3.3.2) package *pheatmap*. Rows represent chromosome arms and are arranged from 1p to 22q from top to bottom. Copy number gains are indicated with red colors; deletions with blue colors, with intensity depicting copy number as indicated in the legend. **B)** Frequencies of copy number alterations in HMM and NHMM groups for MGUS samples. The positive portion of the vertical scale is frequency of gains, while the negative portion is minus the frequency of deletions. The positive and negative portions are also colored red and blue colors respectively on the y-axis.

**Supplementary Figure 7. The clonality index correlation between all HMM samples and those without tetraploidy patients.** Each point represents a CNA event and light reds region represents 95% confidence interval.

**Supplementary Table 1. CNA hotspots in MM**. Chromosome arms are given in the rows and hotspots for gain and deletions are given in the column for HMM and NHMM. Each cell value shows proportion of samples effected by event with cytoband name.

**Supplementary Table 2. Descriptive statistics for MM patients.** Patient characteristics are given for HMM and NHMM samples at MM diagnosis.
