# Supplementary figures and images for: Deciphering the chronology of copy number alterations in Multiple Myeloma
Source: Blood Cancer J. 2019 Mar 26;9(4):39. doi: 10.1038/s41408-019-0199-3 (PMC6435669; doi:10.1038/s41408-019-0199-3)

Supplementary Figure 1

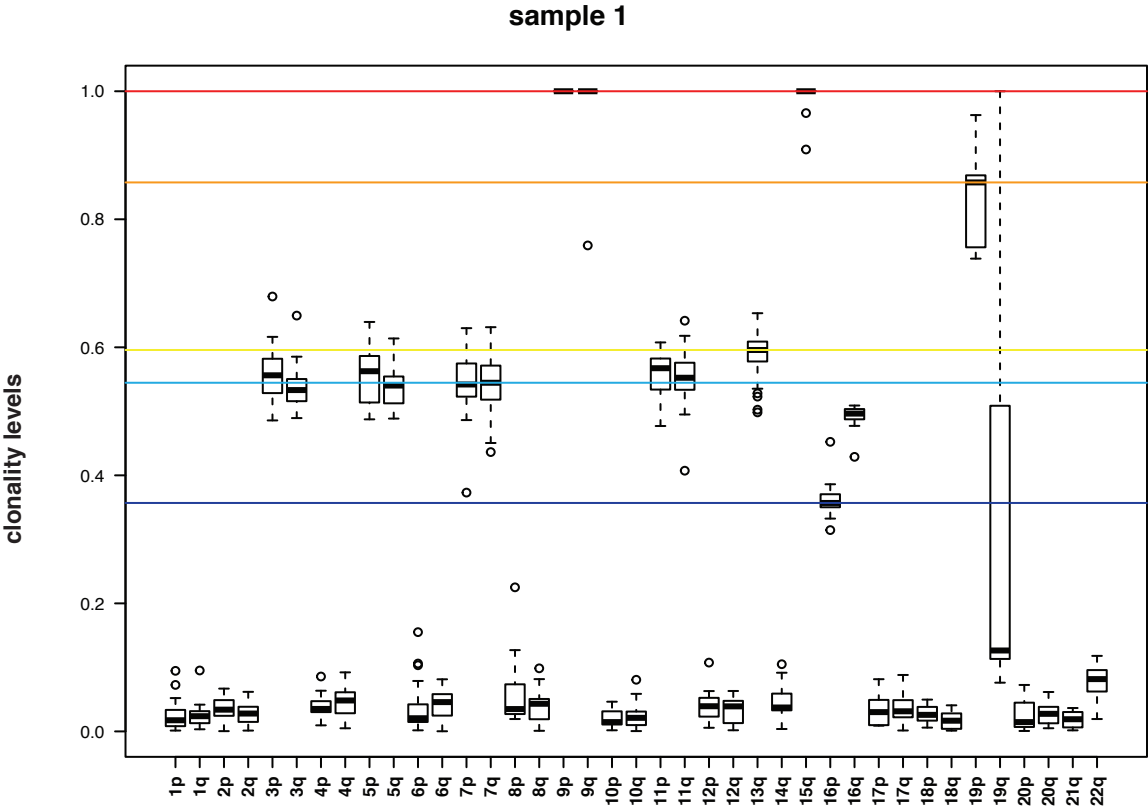

Supplement: Supplementary file 2 — Supplementary Figure 1 [file 41408_2019_199_MOESM2_ESM.pdf]

Supplementary Figure 2

A

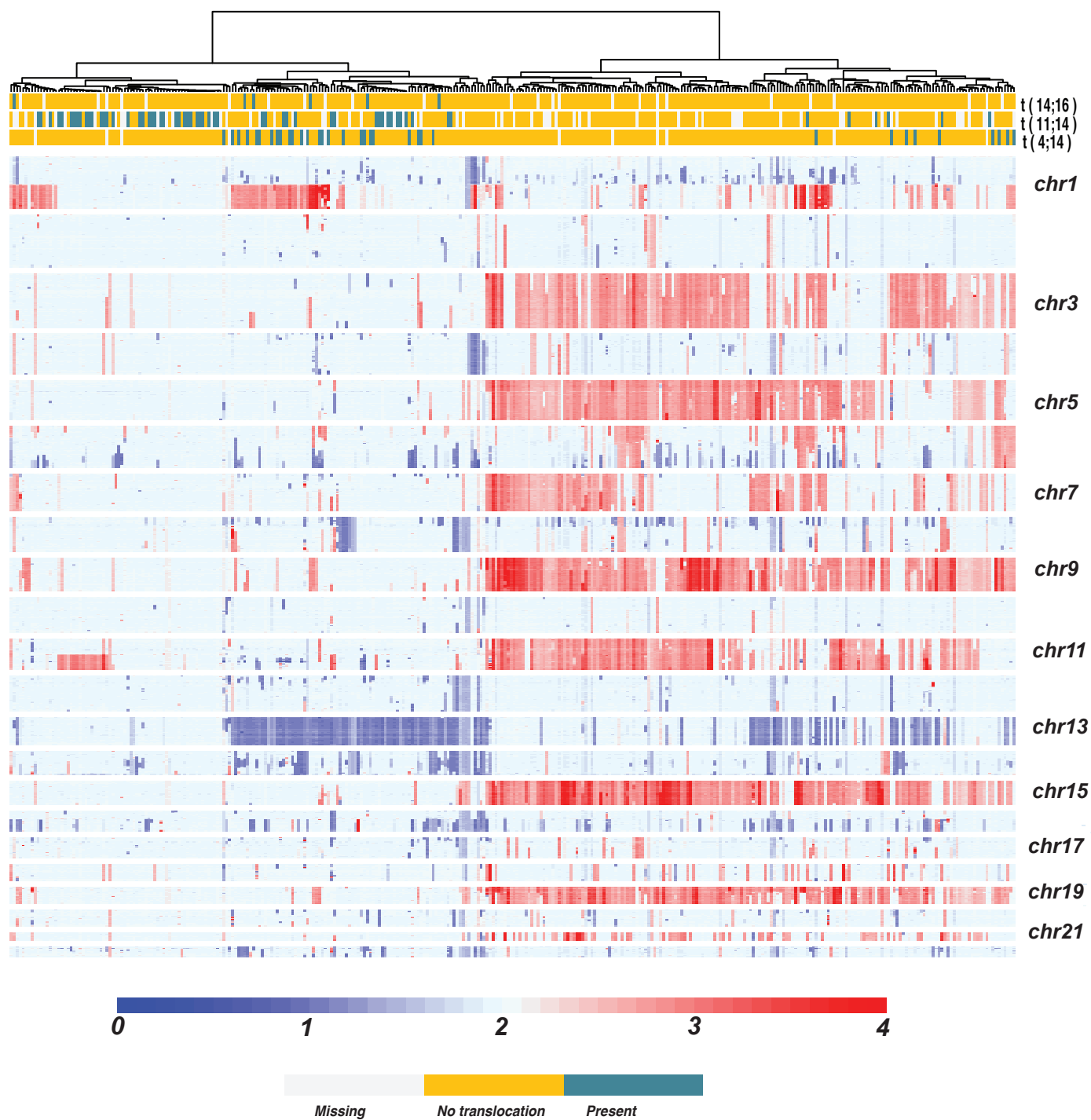

B

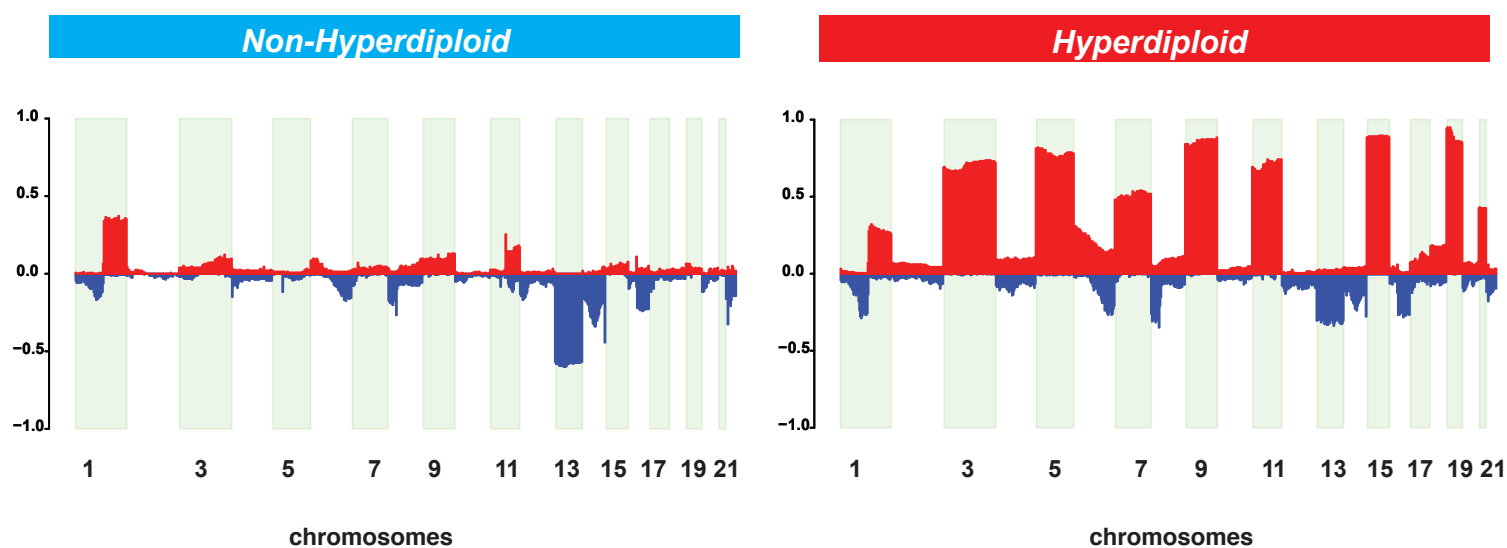

Supplement: Supplementary file 3 — Supplementary Figure 2 [file 41408_2019_199_MOESM3_ESM.pdf]

Supplementary Figure 3

A

MM

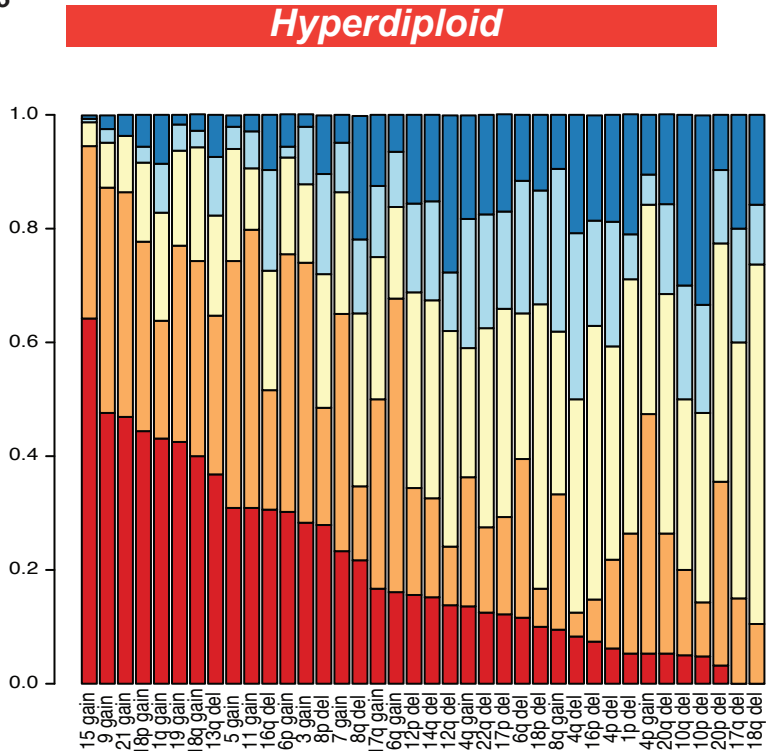

**Non-Hyperdiploid**

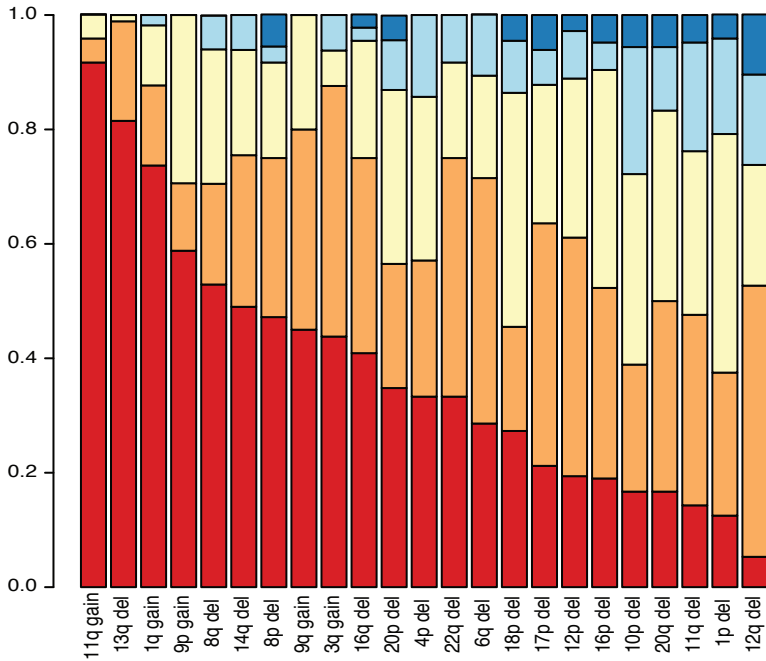

B

MGUS

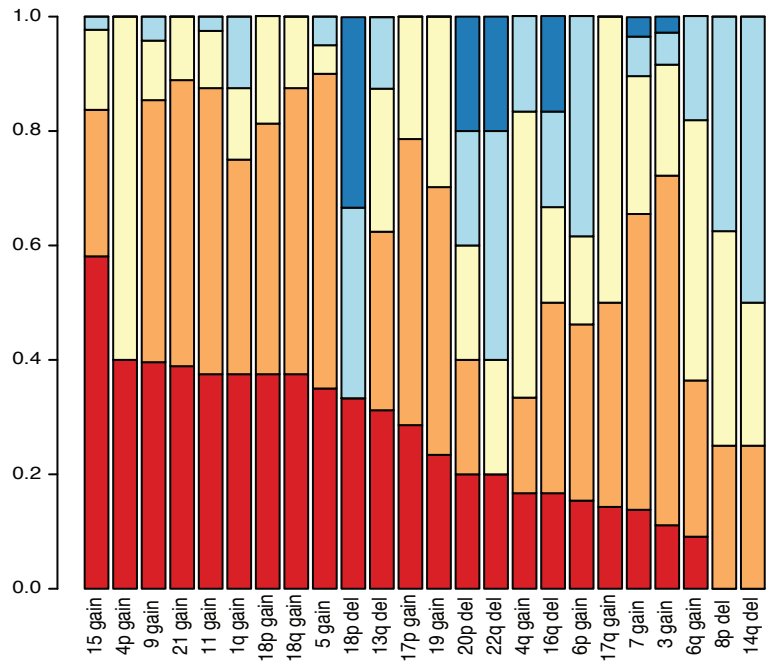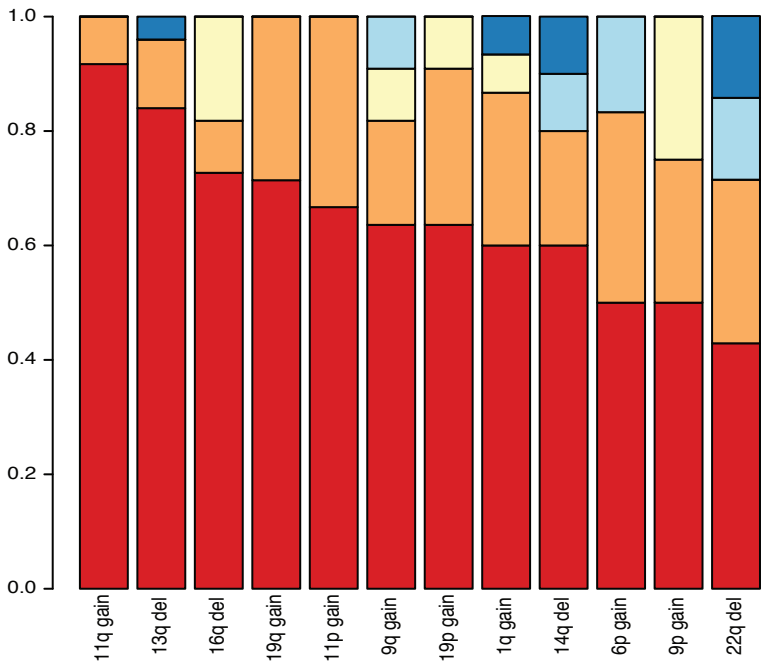

Supplement: Supplementary file 4 — Supplementary Figure 3 [file 41408_2019_199_MOESM4_ESM.pdf]

**Supplementary Figure 4**

**A**

**Hyperdiploid**

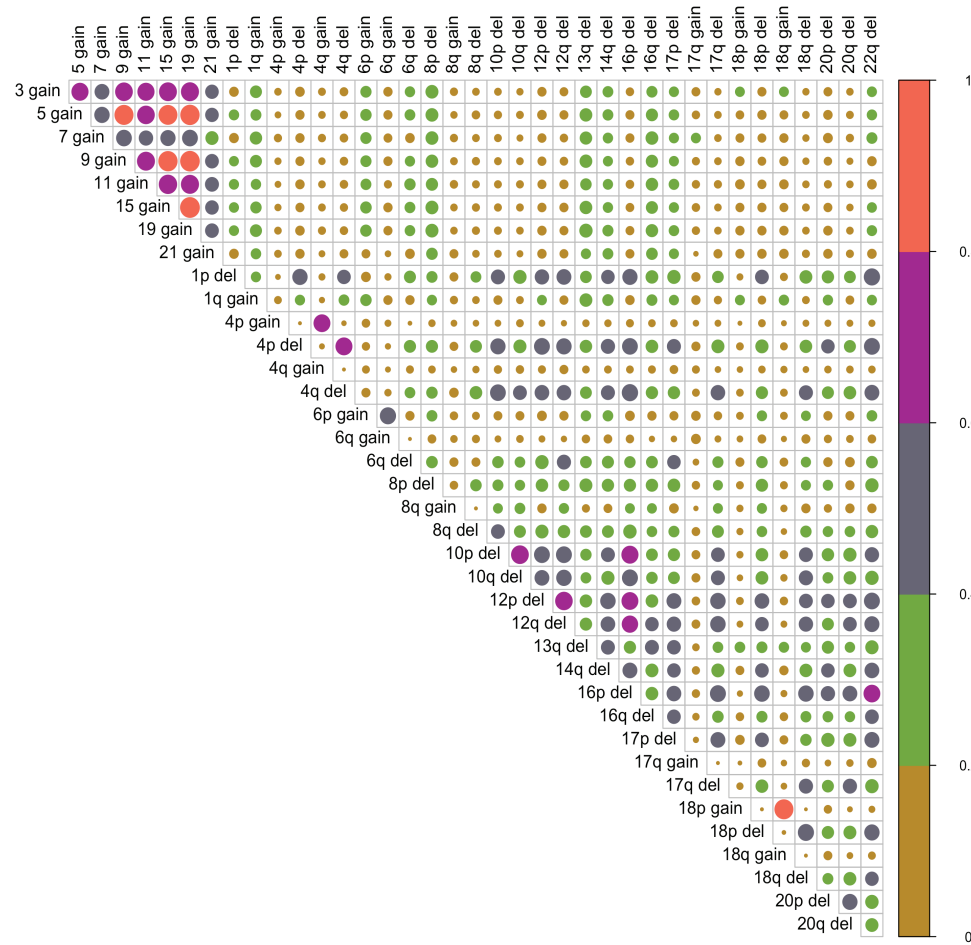

**B**

**Non-Hyperdiploid**

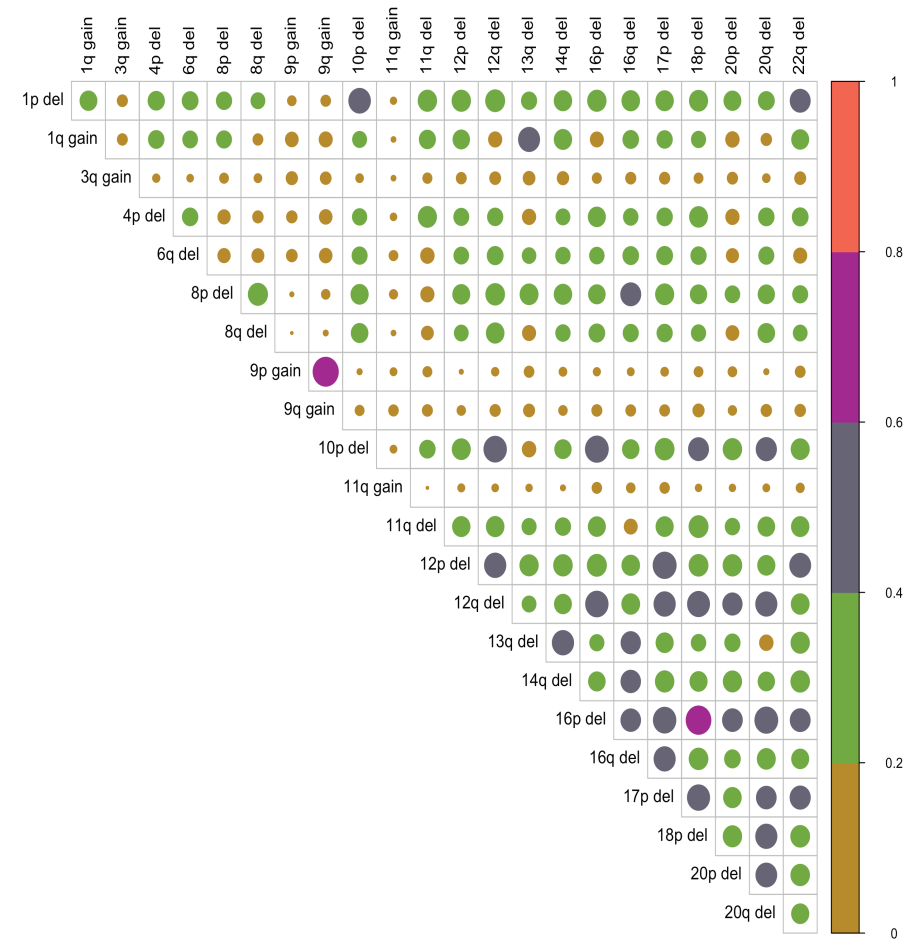

Supplement: Supplementary file 5 — Supplementary Figure 4 [file 41408_2019_199_MOESM5_ESM.pdf]

Supplementary Figure 5

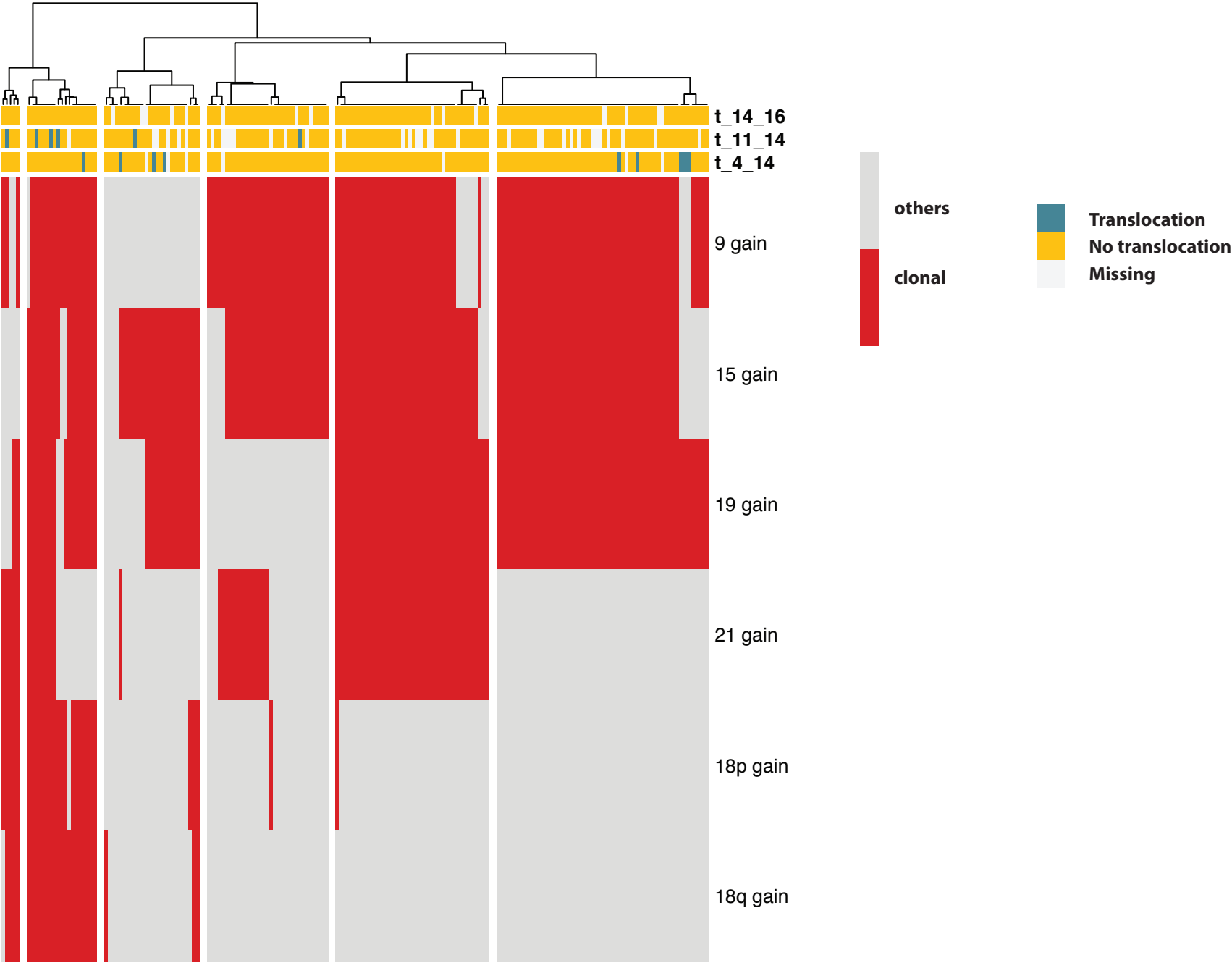

Supplement: Supplementary file 6 — Supplementary Figure 5 [file 41408_2019_199_MOESM6_ESM.pdf]

Supplementary Figure 6

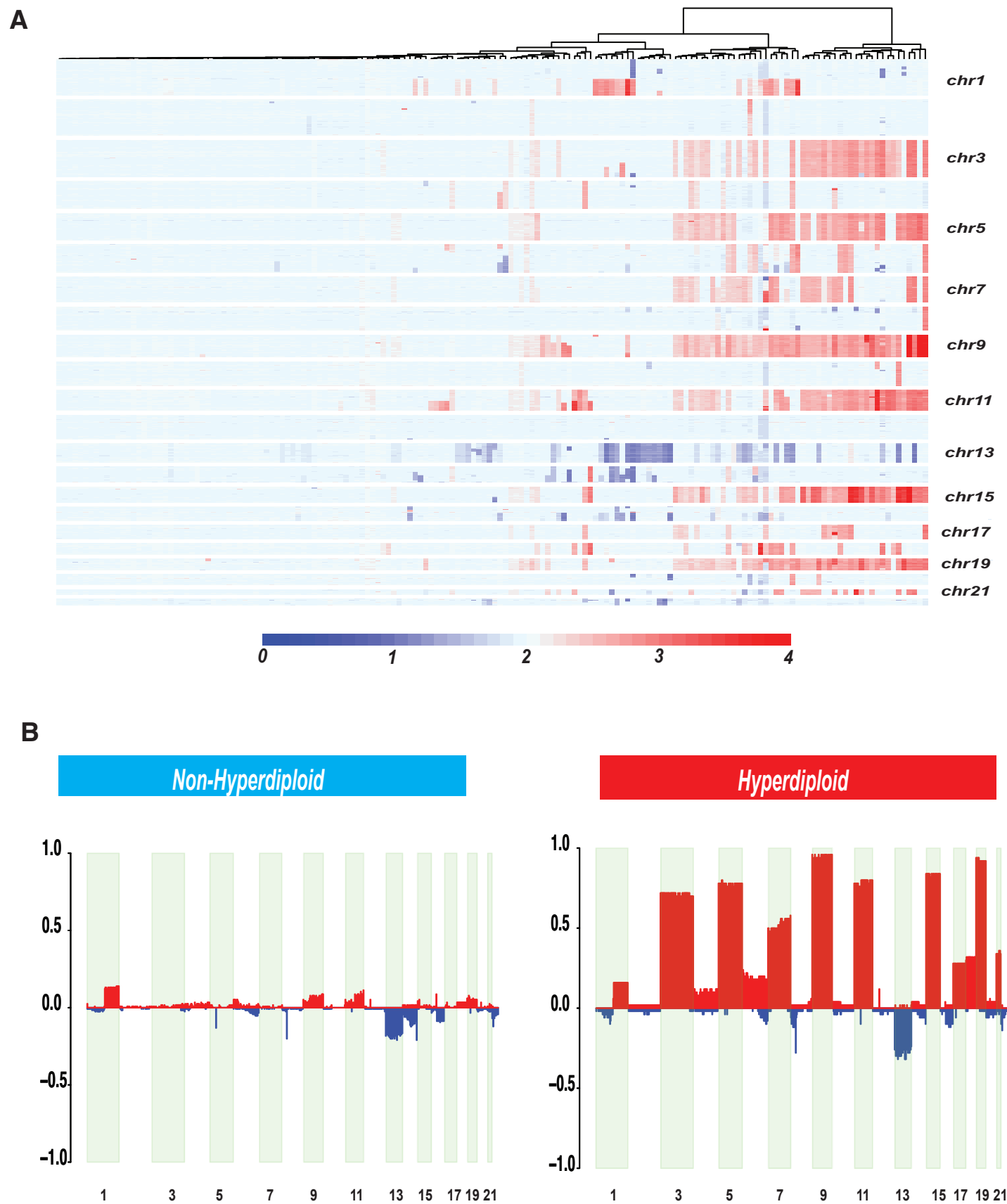

Supplement: Supplementary file 7 — Supplementary Figure 6 [file 41408_2019_199_MOESM7_ESM.pdf]

Supplementary Figure 7

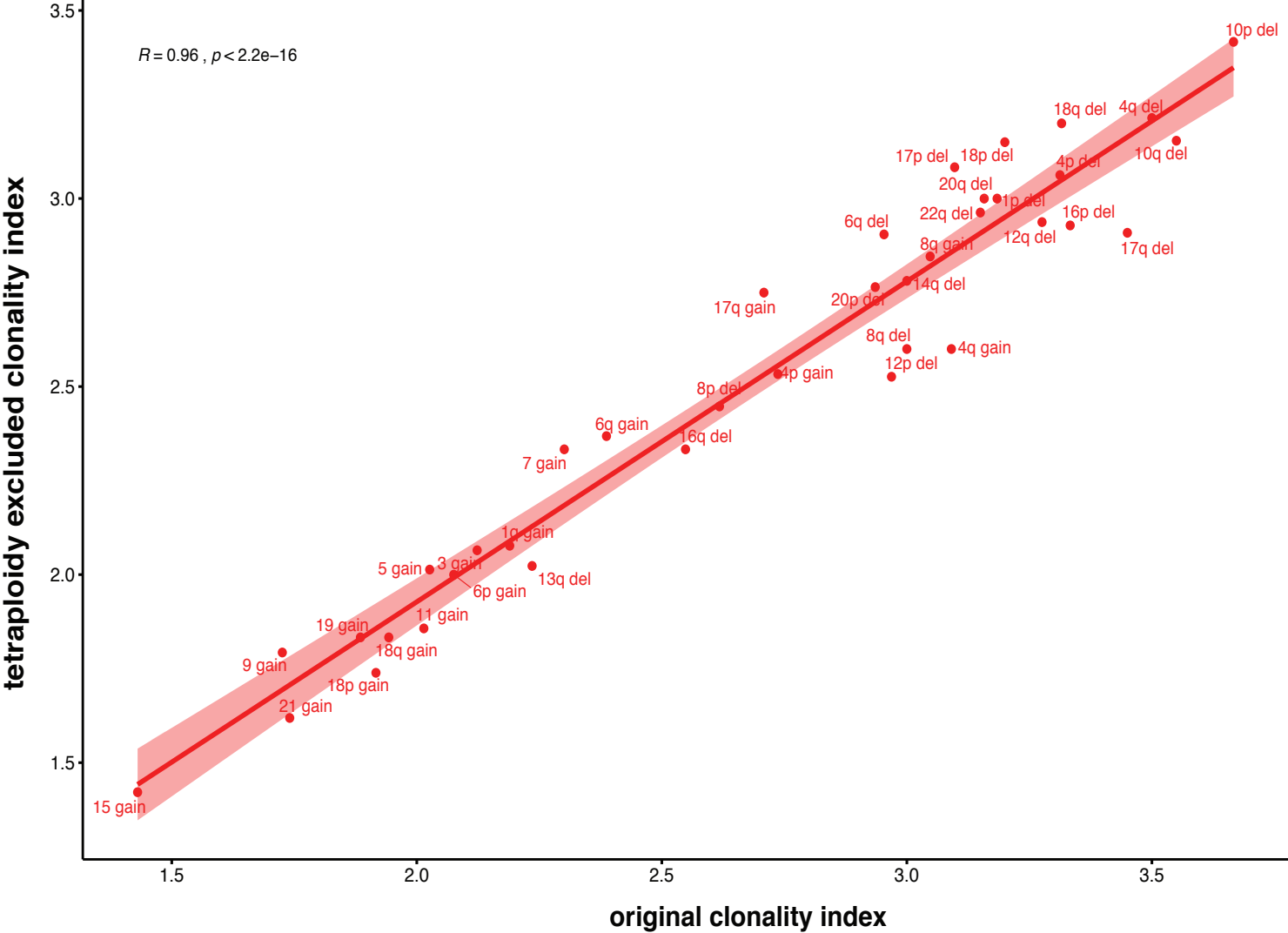

Supplement: Supplementary file 8 — Supplementary Figure 7 [file 41408_2019_199_MOESM8_ESM.pdf]
