# Supplementary material for: Deciphering the chronology of copy number alterations in Multiple Myeloma
Source: Blood Cancer J. 2019 Mar 26;9(4):39. doi: 10.1038/s41408-019-0199-3 (PMC6435669; doi:10.1038/s41408-019-0199-3)
Supplement: Supplementary file 9 — Supplementary Table 1 [file 41408_2019_199_MOESM9_ESM.pdf]

	Hyperdiploid		Non Hyperdiploid	
**Chrosome Arm**	**Peak of loss**	**Peak of gain**	**Peak of loss**	**Peak of gain**
chr1p	0.29-chr1p22.1	0.071-chr1p11.2	0.17-chr1p22.1	0.065-chr1p11.2
chr1p	0.279-chr1p22.2	0.033-chr1p36.32	0.163-chr1p21.2	0.013-chr1p35.3
chr1q	0.038-chr1q24.3	0.322-chr1q22	0.013-chr1q24.2	0.373-chr1q32.2
chr2p	0.049-chr2p24.2	0.071-chr2p25.2	0.026-chr2p24.1	0.046-chr2p25.3
chr2q	0.098-chr2q36.3	0.06-chr2q11.2	0.039-chr2q31.2	0.007-chr2q33.2
chr3p	0.011-chr3p13	0.705-chr3p11.2	0.039-chr3p21.31	0.052-chr3p25.1
chr3q	0.011-chr3q13.11	0.738-chr3q25.32	0.02-chr3q26.31	0.124-chr3q26.2
chr4p	0.109-chr4p15.32	0.098-chr4p12	0.15-chr4p16.3	0.033-chr4p16.3
chr4q	0.142-chr4q21.22	0.109-chr4q35.2	0.046-chr4q11	0.046-chr4q31.3
chr5p	0.011-chr5p15.1	0.82-chr5p15.31	0.118-chr5p11	0.02-chr5p15.33
chr5q	0.011-chr5q11.1	0.787-chr5q32	0.046-chr5q15	0.033-chr5q33.3
chr6p	0.044-chr6p11.1	0.311-chr6p25.2	0.02-chr6p12.2	0.098-chr6p24.3
chr6p	0.027-chr6p25.1	0.306-chr6p25.3	0.02-chr6p12.1/chr6p11.2/chr6p11.2	0.098-chr6p22.3
chr6q	0.268-chr6q24.1	0.213-chr6q11.1	0.176-chr6q24.2	0.02-chr6q11.1
chr6q	0.268-chr6q25.2	0.208-chr6q11.2/ chr6q12	0.170-chr6q23.3 / chr6q24.1	0.02-chr6q11.2/chr6q12 /chr6q13/chr6q14.1/chr6q25.3
chr7p	0.055-chr7p22.2	0.508-chr7p15.2	0.078-chr7p22.3	0.072-chr7p15.2
chr7q	0.022-chr7q35	0.541-chr7q31.2	0.013-chr7q11.22	0.052-chr7q31.2
chr8p	0.35-chr8p11.22	0.071-chr8p11.21	0.268-chr8p11.22	0.033-chr8p23.3
chr8p	0.317-chr8p21.2	0.071-chr8p11.1	0.203-chr8p21.2	0.020-chr8p23.2/chr8p11.21/chr8p11.1
chr8q	0.093-chr8q23.2	0.115-chr8q24.22	0.078-chr8q22.1	0.098-chr8q24.23
chr9p	0.016-chr9p21.3	0.847-chr9p13.3	0.02-chr9p21.3	0.105-chr9p21.1
chr9q	0.005-chr9q13	0.885-chr9q34.3	0.007-chr9q21.11	0.131-chr9q33.1
chr10p	0.049-chr10p14	0.022-chr10p15.3	0.059-chr10p11.23	0.013-chr10p15.3

chr10q	0.071-chr10q24.32	0.049-chr10q26.11	0.026-chr10q26.2	0.013-chr10q23.1
chr11p	0.016-chr11p11.12	0.694-chr11p15.5	0.026-chr11p12	0.026-chr11p15.5
chr11q	0.038-chr11q11	0.743-chr11q13.3	0.118-chr11q22.2	0.255-chr11q13.3
chr12p	0.131-chr12p13.1	0.016-chr12p11.1	0.17-chr12p13.2	0.013-chr12p13.33
chr12q	0.104-chr12q21.2	0.027-chr12q11	0.085-chr12q23.2	0.033-chr12q24.31
chr13q	0.339-chr13q22.2	0.033-chr13q31.2	0.601-chr13q14.12	0-chr13q11
chr14q	0.279-chr14q32.33 (IGH)	0.049-chr14q32.32	0.444-chr14q32.33. (IGH)	0.046-chr14q32.31
chr14q	0.24-chr14q24.2; 0.23- chr14q23.3	0.049-chr14q32.32 ; 0.044-chr14q11.2/ chr14q31.2 /chr14q32.2 /chr14q32.31	0.34-chr14q24.1/2;0.327-chr14q23.3/0.320- chr14q23.2	0.046-chr14q32.31; 0.039-chr14q32.2/ chr14q32.33
chr15q	0.005-chr15q11.2	0.896-chr15q22.31	0.026-chr15q26.2	0.078-chr15q26.3
chr16p	0.126-chr16p11.1	0.06-chr16p11.1	0.098-chr16p13.3	0.111-chr16p11.1
chr16q	0.284-chr16q22.2	0.022-chr16q11.2	0.242-chr16q22.2	0.052-chr16q24.3
chr17p	0.126-chr17p13.1	0.082-chr17p11.2	0.118-chr17p13.1	0.02-chr17p13.3
chr17q	0.071-chr17q24.3	0.142-chr17q22	0.039-chr17q11.1	0.033-chr17q21.32
chr18p	0.082-chr18p11.32	0.186-chr18p11.23	0.046-chr18p11.32	0.033-chr18p11.22
chr18q	0.077-chr18q21.2	0.191-chr18q23	0.033-chr18q12.2	0.039-chr18q21.33
chr19p	0-chr19p13.3	0.951-chr19p13.2	0.026-chr19p11	0.065-chr19p13.3
chr19q	0.005-chr19q11	0.891-chr19q12	0-chr19q11	0.039-chr19q13.13
chr20p	0.115-chr20p12.3	0.077-chr20p11.1	0.118-chr20p13	0.007-chr20p12.3
chr20q	0.082-chr20q13.11	0.077-chr20q13.33	0.046-chr20q13.12	0.039-chr20q13.33
chr21q	0.038-chr21q11.2	0.432-chr21q21.1	0.013-chr21q22.11	0.026-chr21q22.11
chr22q	0.18-chr22q11.22	0.06-chr22q11.22	0.327-chr22q11.22	0.052-chr22q13.1
chr22q	0.137-chr22q12.1	0.033-chr22q11.23/32	0.209-chr22q12.2	0.046-chr22q11.22
