# Supplementary material for: Deciphering the chronology of copy number alterations in Multiple Myeloma
Source: Blood Cancer J. 2019 Mar 26;9(4):39. doi: 10.1038/s41408-019-0199-3 (PMC6435669; doi:10.1038/s41408-019-0199-3)
Supplement: Supplementary file 10 — Supplementary Table 2 [file 41408_2019_199_MOESM10_ESM.docx]

|  | **Overall at  Diagnosis (n=336)** | **HMM (n=183)** | **NHMM (n=153)** |
| --- | --- | --- | --- |
| **Age** | | | |
| ***< 60*** | 177 (53%) | 83 (45%) | 94 (61%) |
| ***>= 60*** | 159 (47%) | 100 (55%) | 59 (38%) |
| **Gender** | | | |
| ***Female*** | 119 (39%) | 59 (35%) | 60 (44%) |
| ***Male*** | 184 (61%) | 108 (65%) | 76 (56%) |
| **Translocations** | | | |
| ***t(4;14)*** | 34 (10%) | 9 (5%) | 25 (16%) |
| ***t(11;14)*** | 59 (18%) | 6 (3%) | 53 (35%) |
| ***t(14;16)*** | 6 (2%) | - | 6 (4%) |
| **ISS** | | | |
| ***I*** | 108 (32%) | 64 (35%) | 44 (29%) |
| ***II*** | 156 (47%) | 88 (48%) | 68 (45%) |
| ***III*** | 69 (21%) | 30 (17%) | 39 (26%) |
